# Novel anchor ring-shaped thread countertraction for colorectal endoscopic submucosal dissection beyond the hepatic flexure

**DOI:** 10.1055/a-2901-6505

**Published:** 2026-07-08

**Authors:** Masahito Kokubu, Hirohito Mori, Yasunori Yamamoto, Makoto Sato, Masaaki Tange, Yoichi Hiasa

**Affiliations:** 1 Department of Gastroenterology 89449 Ehime Rosai HospitalNiihamaEhimeJapan; 2 Department of Advanced and Innovative EndoscopyEhime University Graduate School of MedicineToonEhimeJapan; 3 Department of Gastroenterology and MetabologyEhime University Graduate School of MedicineToonEhimeJapan

**Keywords:** Endoscopy Lower GI Tract, Colorectal cancer, Endoscopic resection (polypectomy, ESD, EMRc, ...)

Endoscopic submucosal dissection (ESD) has become a standard therapeutic option for colorectal neoplasms. However, the technical difficulty of the procedure might depend on its location. In particular, large lesions extending across the hepatic or splenic flexure often prevent a clear operative view. This makes ESD technically challenging. To date, as several traction-assisted techniques have been reported, 1​ 2​ 3​ 4​ ​5​ we introduce a novel countertraction method designed to facilitate ESD in these anatomically complex areas.

**Fig. 1:**
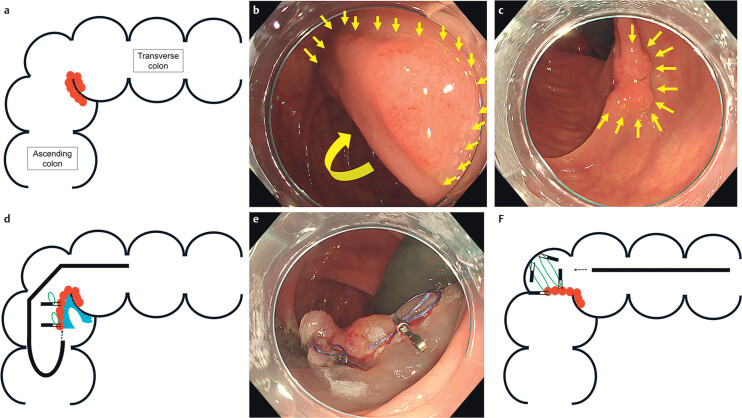
Endoscopic submucosal dissection (ESD) of a large colorectal lesion located across the transverse and hepatic flexure. ( **a** ) Schematic illustration showing the lesion (red) straddling the ascending colon and transverse colon. ( **b** ) An endoscopic view from the anal side: the extent of the lesion is indicated by yellow arrows, with curved arrows suggesting that the lesion extends beyond the mucosal fold. ( **c** ) An endoscopic view from the oral side showing the proximal margin of the lesion. ( **d** ) Schematic illustration showing the endoscope retroflexed within the ascending colon to approach the lesion from its oral side. ( **e** ) Two zeoclips (Zeon Co., Tokyo) equipped with 5-mm ring-shaped threads were applied to grasp the dissected oral edge of the lesion. ( **f** ) Schematic illustration showing the clips anchored to the opposite colonic wall via the ring thread.

An 84-year-old man underwent ESD for a colorectal tumor. The lesion was a 40-mm laterally spreading tumor of the non-granular type extending across the transverse colon and hepatic flexure ( Fig. 1a–c ). ESD was challenging because the lesion was located behind a mucosal fold.

**Fig. 2:**
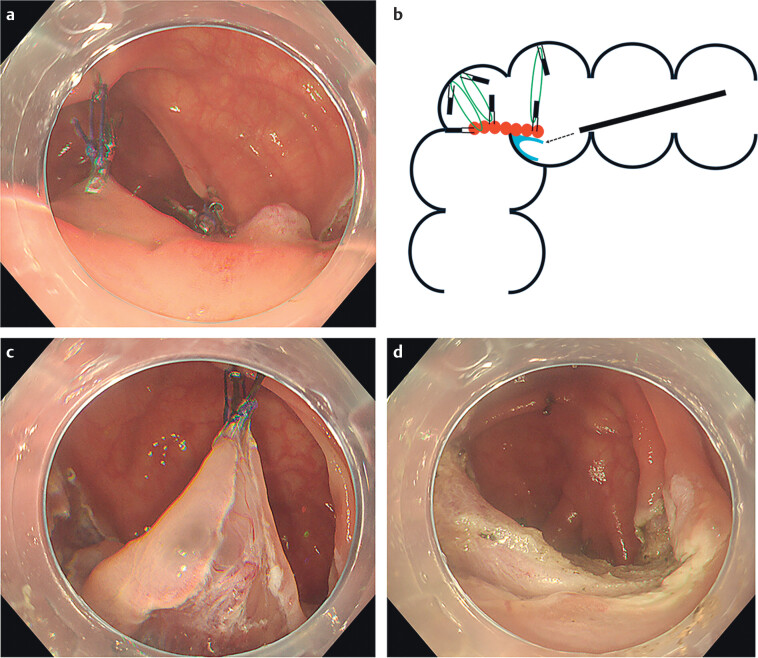
Ring-thread countertraction technique enabling en bloc endoscopic submucosal dissection. ( **a** ) By lifting the oral edge of the lesion using the anchored ring-thread clips, the entire lesion became visible from the anal side, providing a sufficient operative field. ( **b** and **c** ) Schematic illustrations and corresponding endoscopic views showing the ring-thread countertraction technique applied to the anal side of the lesion to facilitate submucosal dissection. ( **d** ) The lesion was successfully resected en bloc.

Initially, the endoscope was retroflexed in the ascending colon to approach the lesion from its oral side, and submucosal dissection was conducted from this side ( Video 1 ). Subsequently, two zeoclips (Zeon Co., Tokyo) equipped with 5-mm ring-shaped threads were applied to grasp the dissected edge ( Fig. 1d,e ). After returning the endoscope to the forward view, another clip was used to anchor the ring threads to the opposite wall of the transverse colon ( Fig. 1f ). This technique effectively straightened the lesion, enabling a stable, direct view of the entire tumor from the anal side( Fig. 2a–c ). With this countertraction in place, submucosal dissection was continued from the anal side, and the lesion was successfully resected en bloc ( Fig. 2d ).

**Video 1** Novel anchor ring-shaped thread countertraction for colorectal endoscopic submucosal dissection of a large laterally spreading tumor across the hepatic flexure.


Unlike dedicated multi-point devices such as ATTRACT, 1​ ​2​ this technique requires only commercially available zeoclips and ring-shaped threads, while the anchor-ring configuration provides panoramic countertraction across the lumen for flexure-crossing lesions. This panoramic countertraction across the lumen is, to our knowledge, a structural feature that has not been described for ATTRACT or other established clip-and-thread traction methods.

Endoscopy_UCTN_Code_TTT_1AO_2AG_3AD
